# Effect of Brief Daily Resistance Training on Occupational Neck/Shoulder Muscle Activity in Office Workers with Chronic Pain: Randomized Controlled Trial

**DOI:** 10.1155/2013/262386

**Published:** 2013-12-31

**Authors:** Mark Lidegaard, Rene B. Jensen, Christoffer H. Andersen, Mette K. Zebis, Juan C. Colado, Yuling Wang, Thomas Heilskov-Hansen, Lars L. Andersen

**Affiliations:** ^1^National Research Centre for the Working Environment, Lersø Parkallé 105, 2100 Copenhagen Ø, Denmark; ^2^Gait Analysis Laboratory, Copenhagen University Hospital Hvidovre, Kettegaard Alle 30, 2650 Hvidovre, Denmark; ^3^Research Group in Sport and Health, Laboratory of Physical Activity and Health, University of Valencia, 46010 Valencia, Spain; ^4^Department of Rehabilitation Medicine, The Sixth Affiliated Hospital of Sun Yat-sen University, No. 26, Yuancun 2nd Cross Road, Guangzhou 510655, China; ^5^Department of Occupational and Environmental Medicine, Bispebjerg University Hospital, Bispebjerg Bakke 23, 2400 Copenhagen NV, Denmark

## Abstract

*Purpose*. This study investigates the acute and longitudinal effects of resistance training on occupational muscle activity in office workers with chronic pain. *Methods*. 30 female office workers with chronic neck and shoulder pain participated for 10 weeks in high-intensity elastic resistance training for 2 minutes per day (*n* = 15) or in control receiving weekly email-based information on general health (*n* = 15). Electromyography (EMG) from the splenius and upper trapezius was recorded during a normal workday. *Results*. Adherence to training and control interventions were 86% and 89%, respectively. Compared with control, training increased isometric muscle strength 6% (*P* < 0.05) and decreased neck/shoulder pain intensity by 40% (*P* < 0.01). The frequency of periods with complete motor unit relaxation (EMG gaps) decreased acutely in the hours after training. By contrast, at 10-week follow-up, training increased average duration of EMG gaps by 71%, EMG gap frequency by 296% and percentage time below 0.5%, and 1.0% EMGmax by 578% and 242%, respectively, during the workday in m. splenius. *Conclusion*. While resistance training acutely generates a more tense muscle activity pattern, the longitudinal changes are beneficial in terms of longer and more frequent periods of complete muscular relaxation and reduced pain.

## 1. Introduction

Since the start of the industrial revolution in the middle of the 19th century, there have been huge social upheaval and massive technological advances, majorly impacting our way of life. This encompasses a more sedentary working life with extensive computer use [1], illustrated by the fact that as much as 41% of European workers use computer for at least a quarter of the working day [2]. This increases the time with static body postures and repetitive movements of the arm, shoulder, and hand, which has been associated with development of musculoskeletal disorders [3, 4]. Concurrently with this tendency, there has been a pronounced increase in the number of computer-related muscular complaints especially in the neck/shoulders [5], and it has been reported that more than 50% of workers using computer at least 15 hours per week develop muscular skeletal symptoms in the upper extremities within their first work year [6]. This has great individual and societal consequences as neck/shoulder pain in white-collar workers has been shown to increase the risk for long-term sickness absence by 35% [7].

Systematic reviews of prospective cohort studies show that gender (woman) and a prior history of neck pain are the strongest predictors for development of neck pain in computer workers [8]. Gender differences may be due to differences in work tasks, work techniques, and women doing more stereotype work tasks relative to men, but also the fact that women in a broad sense at a given work load would have a greater relative exposure due to lower muscle strength [9–12]. Surprisingly, a recent systematic review showed limited or conflicting evidence for work-related physical and psychosocial factors such as duration of computer and mouse use, influence at work, and job demands [8]. However, such epidemiological studies are often based on questionnaires or software-based registrations of computer use but lack physiological measurements, for example, muscle activity patterns.

Tension or activity of the neck/shoulder muscles may play an important role in the development of neck/shoulder pain and can be measured with electromyography during work. The type of activity patterns in the neck/shoulders muscles associated with computer work causes a selective activation of low-threshold motor units with type I muscle fibers. This causes both reduced local blood flow and an accumulation of calcium (Ca^2+^), which can lead to musculoskeletal pain [13, 14]. Previous studies have shown an association between higher trapezius muscle activity and neck/shoulder pain [15, 16]. In particular, the frequency of gaps in trapezius muscle activity, that is, periods with complete motor unit relaxation, seems to be associated with neck/shoulder pain [17, 18]. A recent study has documented a longitudinal association between occupational neck/shoulder muscle activity and the risk for developing pain [19]. Thus, individuals with higher levels of occupational neck/shoulder muscle activity, that is, higher levels of muscle tension, may be at higher risk of developing neck pain. Consequently, interventions to induce a more relaxed muscle activity pattern during work may be beneficial.

Previous research has shown that physical exercise reduces musculoskeletal pain [20–22]. Some studies have investigated the effect of resistance training on neck/shoulder pain. While an acute increase in pain can occur in response to a single bout of high-intensity resistance training at the beginning of the training period in neck pain patients [23], previous studies have shown beneficial long-term effects of resistance training in terms of reduced neck pain [24–26]. Our lab has previously shown that office workers and laboratory technicians experience promising and effective reductions in neck/shoulder/arm pain in response to 10–20 weeks of resistance training with either dumbbells or elastic resistance bands [27–29], and a dose-response analysis indicated that one to two 20 minute training sessions per week appear to be sufficient for pain relief [30]. Importantly, a moderate reduction in pain [28] and muscle tenderness [27] can be obtained in response to as little as two minutes of daily neck/shoulder resistance training performed as a single set to failure. However, the mechanisms of pain reduction in response to such minimal amounts of high-intensity training are unknown. It can be speculated that resistance training causes reductions in the relative muscle force used or altered muscle recruitment patterns during work.

This study investigates the effect of brief daily resistance training on the acute and longitudinal changes in occupational electromyographic activity of the neck muscles (m. splenius and m. trapezius) in female office workers with neck/shoulder pain. We hypothesized that performing two minutes of daily neck/shoulder resistance training for 10 weeks will beneficially alter the muscular activity pattern and thereby reduce neck/shoulder pain. In detail, we hypothesized that the training group will experience (i) an enhanced frequency of EMG gaps, (ii) a prolonged duration of the EMG gaps, and (iii) have a larger percentage of time with a minimal muscular activity compared with the control group.

## 2. Methods

### 2.1. Study Design and Participants

**Figure 1:**
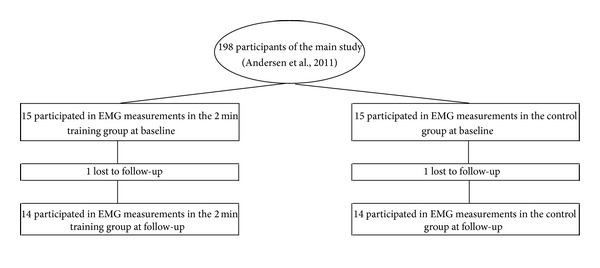
Flow chart.

This study is nested in a larger randomized controlled trial performed in Copenhagen, Denmark. In the larger parallel-group single-blind randomized controlled trial, the participants were allocated to training groups of two or twelve minutes of daily resistance training or to a control group. For the present analyses, we were particularly interested in the mechanisms of pain reduction in the group performing a single set to failure and included a subsample of 2 × 15 participants. In the larger study, 198 office workers with frequent neck/shoulder pain, but without traumatic injuries or serious chronic disease participated. However, due to the time-consuming procedure of performing full-day EMG measurements, it was not possible in the present study to perform daily EMG measurements on all 198 participants. The detailed procedure of recruitment and concealed randomization of the 198 participants is described elsewhere [28]. In brief, the participants recruited were employees from one large office workplace characterized by computer use for the majority (90%) of the working time.  Figure 1 provides an overview of the entire flow of the participants throughout the study. After randomisation, emails were sent to the participants of the larger study inviting them to participate in workday measurements with EMG. When 15 positive replies in each group were obtained, the recruitment was closed. The minimal sample size was estimated on background of data from a prior study on EMG measurements [31]. The recruitment was started during August 2009 and was terminated in September 2009, where the baseline measurements were also conducted. The last participant had follow-up in December 2009.

The outcomes in this nested study of the trial were change in (i) frequency of EMG gaps under 0.5% EMGmax (number per minute), (ii) duration per EMG gap under 0.5% EMGmax (length in seconds), and (iii) time spent under 0.5% EMGmax (percentage distribution). On an exploratory basis, the time spent under 1.0%, 1.5%, and 2.0% EMGmax was also investigated. These outcomes were assessed both acutely after a training session and longitudinally following the 10-week intervention. There were no changes made to either methods or study protocol after trial registration.

All participants were informed about the purpose and content of the study and gave their written informed consent prior to participating in the study, which conformed to The Declaration of Helsinki and was approved by the local ethical committee of Copenhagen and Frederiksberg (HC2008103).

### 2.2. Intervention

The intervention has been described in detail elsewhere [28]. In brief, the training group of the present study performed two minutes of shoulder abductions in the scapular plane with an elastic tubing (Thera-Band) as added resistance on a daily basis on workdays during their working hours. This exercise is also known as “lateral raise” and it effectively targets most neck/shoulder muscles [31, 32]. Participants performed a single set of exercise with as many consecutive repetitions as possible to momentary muscular fatigue (i.e., to failure) for a maximum duration of two minutes. Afterwards, they registered all training activities in a log to allow for a gradual progression in repetitions and resistance. The control group received e-mail-based information once a week during the 10-week intervention period on various aspects of general health (e.g., diet, smoking, alcohol, physical exercise, stress management, workplace ergonomics, and indoor climate).

### 2.3. Adherence

The adherence in both groups was monitored by weekly internet-based questionnaires. Adherence for the training group was defined as the number of completed training session expressed as a percentage of the total number of training sessions throughout the intervention period. The adherence for the control group was defined as the number of read informational emails expressed as a percentage of the total number of informational emails throughout the intervention period.

### 2.4. Experimental Setup

The EMG signal was recorded from m. trapezius and m. splenius of the dominant side. The recordings were collected using a bipolar surface EMG configuration (Ambu Blue Sensor N, N-00-S, Ambu A/S, Ballerup, Denmark) using an interelectrode distance of two cm [33, 34]. Prior to applying the electrode pairs, the skin was abraded to ensure an impedance level less than 10 kΩ. The electrode pairs were placed in accordance with the SENIAM guidelines (http://www.seniam.org/).

Each pair of EMG electrodes was connected to a wireless probe (Velamed Medizintechnik GmbH) connected to the skin, serving as reference electrode. Furthermore, the probe preamplified the EMG signal (gain 400) before transmitting the data to 16-channel 16 bit PC-interface receiver in real-time (Noraxon Telemyo DTS Telemetry, Noraxon, AZ, USA). All data were collected using a sample rate of 1500 Hz within a bandwidth of 10–500 Hz. This wireless EMG-system has shown to be valid and reliable for collecting EMG-data from the neck/shoulder musculature [35, 36] as well as other muscular groups [37–39].

### 2.5. Experimental Procedure

All EMG recordings were performed during normal working hours while the participants performed their usual work. To obtain resting EMG at the beginning of the workday, participants performed 30 seconds of instructed seated rest with closed eyes and complete arm support while focusing on completely relaxing the shoulder and neck muscles. This was followed by the three reference tasks performed in accordance with outlined guidelines [40]. While seated, the participants held their arms straight and horizontal in 90 degree abduction, the hands were relaxed and palms faced downwards with no additional weight added for a period of 20 seconds [18, 41]. After conducting the reference tasks, the participants were instructed to perform their usual work. After a period of between 60 to 90 minutes the participants conducted an identical reference task. Hereafter, the control group resumed their normal work, while the training group performed their daily training session consisting of two minutes elastic resistance training before resuming their normal work.

**Figure 2:**
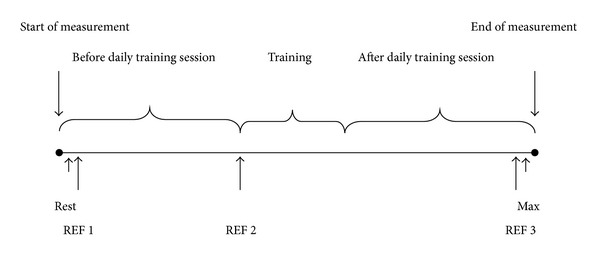
A schematic overview of the measurement period. Rest is equivalent to the resting period where the resting EMG amplitude was determined, REF is equivalent to the three reference tasks, and Max corresponds to the time of the maximal contraction.

After another period of between 60 to 90 minutes just before terminating the measurement, the participants again conducted the reference task. This was followed by a resisted maximal voluntary contraction to obtain maximal EMG for normalization of the obtained EMG signals. The maximal contraction was conducted in the position of the reference tasks with the only addition of an opposing force provided by the test instructor. The participants then performed isometric maximal voluntary contractions two times for five seconds separated by rest periods of 30 seconds. For an overview of the sampling protocol see Figure 2.

### 2.6. Data Collection Area and Recording Time

In the baseline screening questionnaire, the participants reported that they spend the vast majority of their working hours doing computer work, see Table 1. Therefore, prior to each measurement, a data collection area was defined which only included the nearest area around the primary workstation of the participant. This would cause the EMG probes to stop recording data when the participants were not present in the predefined data collection area and thereby automatically filtering out periods where the employees performed other types of activities than their main job function, see Table 2.

### 2.7. Processing of Data

All data processing was performed in MatLab (MathWorks, version 7.5.0 342, R2007b). The first step in the data processing was to filter out the periods of work time were the participants were outside the predefined data collection area. In the measurements, this was visualized as a completely flat line without fluctuations of EMG amplitude, and the program therefore removed periods which assumed identical values over a period of minimum 100 ms.

There were no statistical differences regarding the total recording time and the computer work time between the two groups, see Table 2. For a detailed overview of the relationship between total recording time and the effective time that the participants were located within the predefined data collection area, see the EMG signal which was normalized by determining the maximal Root Mean Square (RMS) during the isometric maximal voluntary contraction. RMS was determined using a moving window with a width of 1500 data points (i.e., 1 sec) and a movement of 100 ms [42]. Subsequently, the resting EMG amplitude was determined, by identifying the lowest RMS within a time period of five seconds during the resting period. The lowest RMS value was quadratically subtracted from all other EMG signals [43]. Hereafter, the RMS plots for both the maximal contraction and the relaxation measurement were visually controlled for 50 Hz interference, unilateral spikes, and abnormalities in the EMG signal.

Finally, the RMS for the working periods before (first 60–90 minutes of data sampling) and after the daily training session (last 60–90 minutes) was determined, using the same procedure as described above. This allowed the identification of periods where the EMG amplitude was below a predefined percentage of the normalized EMGmax, which was termed an EMGgap. In this study, the following percentages of the normalized EMGmax had a particular interest: 0.5%, 1.0%, 1.5%, and 2% EMGmax. According to previous studies, 0.5% EMGmax represents the boundary for total relaxation of a motor unit, whereas the remaining values represent different degrees of activation of the smallest motor units [41]. However, all periods with a very low level of muscle activity up to 2% of maximal EMG had a particular interest. In order to be classified as an EMGgap, the EMG amplitude additionally had to be below 0.5% EMGmax for a period of at least 0.2 s [44, 45].

### 2.8. Statistical Analysis

All statistical analyses were performed in SAS statistical software (SAS version 9.2, SAS Institute, Cary, NC) and were performed in accordance with the intention-to-treat principle by including data from all available participants regardless of actual adherence [46]. Muscle strength and pain were analysed using parametric statistics and reported as mean (SD). However, a Shapiro-Wilk test showed that EMG data generally did not fit a normal distribution. Therefore, we used nonparametric statistics, Mann-Whitney *U* test, to determine between-group differences in all EMG parameters and reported data as medians (interquartile range). All comparisons were performed two-tailed and a probability level of *P* < 0.05 was considered to indicate significant differences.

## 3. Results

Table 1 gives an overview of the characteristics in the two intervention groups at baseline and shows that the groups were matched for demographic, clinical, and work related characteristics.

During the intervention period, the training group performed an average of 4.3 of the 5 scheduled training sessions per week, which is equivalent to an 86.8% training adherence, while the control group had read on average 8.9 of the 10 informational emails corresponding to an adherence of 89%.

Overall, two participants were lost to follow-up, one participant in each intervention group, both due to lack of time. No adverse events were reported during the intervention or EMG measurements.

### 3.1. Recording Time

Table 2 displays the relationship between the total recording time and the effective time the participants were located within the predefined data collection area. As shown in the table, there was no difference in the total sampling time between the intervention groups at either week 0 or week 10. Furthermore, there were no differences within each intervention group at either week 0 or week 10.

### 3.2. Acute Effect of Training

Table 3 shows the frequency of EMG gaps (number per minute). The training group significantly decreased the frequency of EMG gaps in m. splenius by almost 35% from 12.3 to 8.0 gaps/minute acutely in response to the training session at follow-up (*P* < 0.05), that is, an acute worsening of the muscle activity pattern.

### 3.3. Effect of the 10-Week Intervention

Table 3 shows the frequency of EMG gaps. Compared with the control group, the training group significantly increased the number of EMG gaps after 10 weeks of training in m. splenius by approximately 300% from 3.1 to 12.3 gaps/minute (*P* < 0.05), that is, a more relaxed muscle activity pattern.

Table 4 shows baseline and follow-up values for pain intensity and muscular strength for both intervention groups. After the intervention period, the training group significantly decreased neck/shoulder pain intensity by 40% compared with the control group (*P* < 0.01). Furthermore, the training group improved muscular strength by 6%, which was significant compared with the control group (*P* < 0.05).

Tables 5(a) and 5(b) show the percentage distribution of time spent under different levels of EMGmax for m. trapezius and m. splenius, respectively. After 10 weeks of training, there were a significant increase in the percentage of time spent under both 0.5% (*P* < 0.01) and 1.0% (*P* < 0.05) EMGmax in m. splenius for the training group when compared with the control group, from 2.3% to 15.6% and from 7.6% to 26.0%, respectively, corresponding to a 575% and 242% increase in time.

Table 6 shows the average duration in seconds per EMG gap. Compared with the control group, there was a significant increase in the average duration per gap in both m. trapezius and m. splenius for the training group after 10 weeks of training (*P* < 0.05 and *P* < 0.01, resp.) from 0.72 sec to 1.26 sec and from 0.42 sec to 0.72 sec, respectively, corresponding to a 75% and 71% increase, that is, longer periods with complete relaxation.

### 3.4. Reference Contraction

There was no change in the average EMG amplitude during the reference contraction (i.e., arms 90 degree abducted) from before to after the daily training session, showing that the EMG measurements were stable throughout the day.

## 4. Discussion

The main finding of the present study was the change in occupational neck muscle activity in response to brief daily resistance training. These alterations were shown both acutely in response to a single training session and longitudinally following the 10 week intervention—however with opposite impact on the muscle activity pattern. While the single training session acutely altered the muscle activity pattern so that less frequent periods of muscular relaxation were observed, the longitudinal change in muscle activity led to both longer and more frequent periods of complete muscular relaxation. The longitudinal changes were observed concurrently with increased muscle strength and reduced pain of the neck muscles.

### 4.1. Acute Worsening

The frequency of EMG gaps decreased immediately after the training session in the splenius muscle, which may lead to increased muscle tension and perceived discomfort. Although we did not measure acute changes in pain in the present study, previous research has reported an acute increase in muscular pain immediately after high-intensity resistance training in women with trapezius myalgia [23]. However, in that study, the acute aggravation of muscular pain disappeared within two hours and the participants experienced an overall pain reduction following a 10-week training period [23]. Our study suggests that the previously observed acute aggravation of pain may be related to the acute increase in muscle tension immediately after resistance training. These results also highlight the importance of explaining to patients that their pain may acutely worsen in response to high-intensity resistance training, but improve in the long term. This may have important practical implications for adherence to the training program. As an alternative explanation of the present findings, EMG amplitude may be artificially increased immediately after training due to increased blood flow. That is, increased blood flow results in an accumulation of liquids and electrolytes in the active muscles, which may improve the conductivity of the electrical signal and thereby increase the EMG amplitude without an actual increase in muscle tension. A possible reason for this phenomenon to only have an impact after the intervention period may primarily be due to the higher intensity by which the resistance training was performed, leading to higher postexercise hyperemia.

### 4.2. Longitudinal Improvement

The 10-week training period led to decreased pain and increased muscular strength in the neck/shoulder muscles. This is in accordance with the main study including all 198 participants [28]. As a possible explanatory mechanism for the observed pain reduction, we found a number of potentially beneficial changes in neck muscle activity. Previous studies have shown that sustained muscular activity in trapezius muscle is a risk factor for developing neck pain [15, 19]. Furthermore, former studies have shown that muscular activity less than 0.5% EMGmax represents total muscular relaxation and less than 2.0% EMGmax represents sole activation of the smallest motor units [41]. Henneman's size principle and the Cinderella Hypothesis state that the motor units with the lowest threshold will create the majority of muscle tension during sustained low intensity work tasks [47, 48]. Thus, the same motor units will remain active throughout the workday regardless of a reduced relative work strain and increased muscular strength. Therefore, the threshold of 0.5% EMGmax—representing complete muscular relaxation—is relevant when trying to avoid prolonged strain of the smallest motor units.

Our study showed increased frequency of EMG gaps, that is, periods with complete muscular relaxation, defined as muscular activity below 0.5% EMGmax, following 10 weeks of resistance training. This more relaxed activity pattern in the neck muscles is likely to reduce fatigue and pain. Additionally, increased duration of EMG gaps in both m. splenius and m. trapezius was found. Prolonged duration of EMG gaps leads to longer episodes of complete muscular relaxation, which potentially can reduce the pain in the neck/shoulder muscles. A possible explanation for this relationship between the length of the EMG gap and the level of pain intensity can be that shorter EMG gaps, compared to longer EMG gaps, cause a higher average work strain [49]. In addition, previous research has found a positive association between pain in the neck/shoulder muscles and EMG gap length [18, 50]. The study by Blangsted showed that pain free subjects experienced EMG gaps of longer duration compared with subjects who suffered from neck/shoulder pain [50]. Furthermore, increased muscular activity in m. trapezius has been linked to trapezius myalgia [16]. Altogether, these studies support the potential effect of the prolonged and more frequent EMG gaps as a possible explanatory mechanism for the reduced neck/shoulder pain observed in the present study.

Rosendal and coworkers have shown that women suffering from chronic neck muscle pain experience increased levels of both lactate and pyruvate in the interstitium as a result of low-force repetitive work [51, 52]. This has been suggested to be a reflect increased anaerobic metabolism related to a reduced blood flow as a consequence of an insufficient capillarization of the muscle fibres. This is supported by findings showing impaired blood flow to the active muscles in people suffering from myalgia [53, 54]. In the present study, the underlying physiological explanation between increased frequency and duration of EMG gaps and decrease of neck pain may partly be due to enhanced blood flow and thereby increased oxygenation and less anaerobic metabolism due to better muscular relaxation. In a previous study, Kadi and coworkers reported enhanced blood flow as result of an improved capillarization with corresponding decrease in muscular pain after a period of specific resistance training [55]. Thus, as another explanation for the present findings, participants in the training group may have experienced a combination of greater improved capillarization combined with a more relaxed muscle pattern, which together allows for enhanced blood flow and thereby oxygen to the active muscles.

In general, the findings of the present study suggest that the splenius muscle compared with trapezius is the primary site for pain sensation in the neck/shoulder muscles due to the fact that EMG alterations primarily appear in the splenius. This is supported by findings of a higher prevalence of severe tenderness in the neck extensors compared with trapezius [56]. This could have practical implication when treating people who suffer from pain in the neck/shoulder muscles, including trapezius myalgia, by having a greater focus on the state of the neck extensors and not only the trapezius muscle. However, more research is needed to determine whether pain in the neck/shoulders is related more strongly to the splenius than the trapezius.

### 4.3. Limitations

A limitation to the present study is that participants could not be blinded due to the general design with a designated training group. This introduces multiple risks of nonspecific effects including possible placebo effects in respect to changes in perceived pain [57, 58] as well as the possibility of a Hawthorne effect [59]. However, it should be noted that the testers, besides the second reference measurement, only interacted with the participants at initiation and termination of the measurements and therefore had no contact with the participants during the time of the measurements, which likely minimize any possible Hawthorne effect. Furthermore, the present study used objective measures of muscle activity during the working day, minimizing both the potential for placebo and Hawthorn effects to act on EMG measurements. Thus, if the muscle activity pattern did change over time, it is unlikely that this is caused by the participants not being blinded to the intervention.

The relatively small sample size increases the risk for statistical type II errors, that is, not finding a significant difference when there is in fact a difference. On the other hand, the lack of Bonferroni correction will increase the risk for statistical type I errors. However, performing a Bonferroni correction will increase the risk of type II errors [60]. On this background, the Bonferroni correction is often considered as being rather conservative and the decision whether to use a Bonferroni correction or not is therefore a matter of balancing the pros and cons. Bonferroni corrections are appropriate when outcome measures are completely random, for example, throwing a dice. However, as this study had predefined hypotheses, the use of Bonferroni correction appears inappropriate.

The use of surface EMG to determine the muscular activity patterns is sensitive to a number of different parameters including electrode placement [61, 62] and the interelectrode distance [63]. Furthermore, crosstalk from the surrounding musculature has a potential to impact on the EMG [64, 65]. However, this should not affect the interpretation of the findings due to the use of recommended procedures when performing surface EMG [66] as well as prior literature has shown that it is possible to differentiate the EMG signal from m. splenius and m. trapezius [67].

## 5. Conclusion

The primary objective of this study was to investigate whether a brief daily resistance training session would have an effect on the muscular activity pattern of the neck/shoulder muscles. In respect to our hypothesis, we reported beneficial long-term changes in both the frequency and duration of the EMG gaps alongside with alterations in the time with minimal muscular activation. In summary, the acute response to a single session of resistance training appeared to generate an unfavourable muscle activity pattern. By contrast, the longitudinal changes were beneficial in terms of longer and more frequent periods of complete muscular relaxation and reduced pain; however, these findings were more pronounced in m. splenius compared to m. trapezius. Future studies on neck/shoulder pain should consider focusing also on the splenius rather than the trapezius alone.

## Figures and Tables

**Table 1:** Baseline characteristics, Mean ± SD. No significant differences were observed.

	Training (*N* = 15)	Control (*N* = 15)
Age (years)	41.7 ± 10.8	40.5 ± 7.27
Height (cm)	168.8 ± 6.68	166.1 ± 4.44
Weight (kg)	66.5 ± 9.07	65.2 ± 10.1
BMI (kg*·*m^2^)	23.3 ± 2.87	23.6 ± 3.58
Pain intensity previous 3 weeks (Scale 0–10)	3.44 ± 1.40	3.24 ± 1.37
Systolic BP (mmHg)	125 ± 12.3	127 ± 15.2
Diastolic BP (mmHg)	86 ± 8.37	84 ± 9.22
Isometric muscle strength (Nm)	41.1 ± 6.71	37.6 ± 13.21
Computer use (% work time)	98.4 ± 6.25	95.0 ± 10.4
Weekly working time (hours)	38.2 ± 3.9	37.0 ± 3.47
Duration of office work (Years)	10.3 ± 8.6	11.7 ± 8.9

**Table 2:** Recording time corresponding to the average number of total minutes recorded. Effective recording time corresponding to the percentage time where the participants were present in the predefined data collection area, that is, percentage of the total minutes recorded included in the data analysis, median (interquartile range). No significant differences were observed.

		Training (*N* = 15)	Control (*N* = 15)
Recording time (min)			
Before daily training session	Week 0	60.0 (42.0–69.1)	64.5 (49.8–79.2)
	Week 10	72.1 (65.3–77.4)	75.5 (56.8–87.8)
After daily training session	Week 0	82.7 (70.1–96.2)	60.9 (47.9–74.4)
	Week 10	62.3 (39.6–82.6)	59.7 (46.7–73.2)
Effective recording time (%)			
Before daily training session	Week 0	79.7 (70.8–92.9)	84.2 (71.9–95.1)
	Week 10	89.3 (84.3–92.5)	93.6 (40.9–97.3)
After daily training session	Week 0	92.2 (73.4–97.0)	93.3 (87.9–95.1)
	Week 10	93.7 (85.6– 98.5)	94.3 (73.5–97.1)

**Table 3:** Frequency of EMG gaps (periods per minute below 0.5% EMGmax) for m. trapezius and m. splenius, median (interquartile range).

		Training (*N* = 15)	Control (*N* = 15)
Trapezius			
Before daily training session	Week 0	8.5 (4.4–11.6)	4.2 (1.9–11.5)
	Week 10	8.2 (6.3–15.3)	3.7 (1.1–13.2)
After daily training session	Week 0	7.4 (5.4–11.7)	3.0 (2.0–12.2)
	Week 10	7.6 (4.4–15.2)	2.2 (1.2–6.4)
Splenius			
Before daily training session	Week 0	3.1 (1.4–10.9)	5.0 (1.6–10.9)
	Week 10	12.3 (4.8–15.2)^b^	1.1 (0.5–5.8)
After daily training session	Week 0	5.0 (2.7–7.8)	3.1 (1.3–11.7)
	Week 10	8.0 (3.5–14.5)^d^	1.3 (0.5–6.5)

^b^ *P* < 0.05 significant change from baseline to follow-up in the training group compared with the control group. ^d^ *P* < 0.05 significant change from before to after the daily training session.

**Table 4:** Pain intensity and muscular strength at week 0 and week 10, Mean ± SD.

		Training (*N* = 15)	Control (*N* = 15)
Pain intensity (scale 0–10)	Week 0	3.44 ± 1.40	3.24 ± 1.37
	Week 10	2.04 ± 1.60^a^	3.45 ± 1.99
Isometric muscle strength (Nm)	Week 0	41.1 ± 1.7	37.6 ± 3.4
	Week 10	43.2 ± 1.3^b^	36.5 ± 3.3

^a^ *P* < 0.01 significant change from baseline to follow-up in the training group compared with the control group. ^b^ *P* < 0.05 significant change from baseline to follow-up in the training group compared with the control group.

**Table:** (a)

			Training (*N* = 15)	Control (*N* = 15)
Trapezius	0.5% EMGmax			
	Before daily training session	Week 0	18.5% (5.1–39.1)	11.4% (4.3–17.6)
		Week 10	24.0% (16.0–34.6)	4.1% (0.9–27.8)
	After daily training session	Week 0	15.1% (11.1–30.2)	9.8% (3.1–12.7)
		Week 10	25.8% (13.4–42.2)	4.9% (1.5–7.9)
	1.0% EMGmax			
	Before daily training session	Week 0	39.0% (14.1–50.8)	21.3% (7.6–34.1)
		Week 10	32.3% (24.3–54.8)	9.9% (2.9–35.5)
	After daily training session	Week 0	25.9% (21.2–41.2)	12.6% (6.3–26.0)
		Week 10	37.0% (20.7–54.3)	6.6% (3.9–13.9)
	1.5% EMGmax			
	Before daily training session	Week 0	47.3% (24.3–57.3)	28.3% (10.2–43.9)
		Week 10	38.6% (32.3–66.1)	19.5% (5.1–40.4)
	After daily training session	Week 0	36.1% (29.1–50.6)	15.2% (10.5–33.3)
		Week 10	46.2% (26.5–62.8)	11.0% (5.7–18.8)
	2.0% EMGmax			
	Before daily training session	Week 0	55.0% (37.6–61.9)	34.7% (15.6–52.3)
		Week 10	44.5% (39.2–72.9)	27.9% (7.3–47.7)
	After daily training session	Week 0	44.9% (35.8–58.3)	19.5% (14.2–39.4)
		Week 10	53.6% (32.9–74.7)	14.4% (7.8–28.5)

**Table:** (b)

			Training (*N* = 15)	Control (*N* = 15)
Splenius	0.5% EMGmax			
	Before daily training session	Week 0	2.3% (1.0–20.7)	7.1% (3.8–11.1)
		Week 10	15.6% (11.7–28.5)^a^	0.8% (0.2–4.6)
	After daily training session	Week 0	5.0% (2.2–8.9)	4.1% (1.3–10.4)
		Week 10	14.3% (8.5–20.0)	1.9% (0.3–5.9)
	1.0% EMGmax			
	Before daily training session	Week 0	7.6% (5.8–35.1)	11.1% (6.4–24.7)
		Week 10	26.0% (21.9–45.0)^b^	3.5% (1.4–12.6)
	After daily training session	Week 0	11.9% (4.3–19.4)	5.9% (2.0–22.5)
		Week 10	23.9% (12.3–29.8)	3.7% (1.0–12.4)
	1.5% EMGmax			
	Before daily training session	Week 0	18.2% (11.1–48.5)	21.0% (7.6–29.8)
		Week 10	37.2% (29.8–55.4)	7.3% (2.8–20.8)
	After daily training session	Week 0	24.6% (10.1–37.6)	8.2% (4.6–32.5)
		Week 10	34.2% (19.2–40.8)	5.7% (2.5–18.8)
	2.0% EMGmax			
	Before daily training session	Week 0	30.1% (19.8–57.9)	31.0% (10.7–37.8)
		Week 10	46.0% (36.2–62.6)	10.6% (5.4–29.3)
	After daily training session	Week 0	35.1% (19.9–51.2)	11.9% (8.8–35.5)
		Week 10	41.0% (29.8–52.8)	9.7% (4.3–26.5)

^a^ *P* < 0.01 significant change from baseline to follow-up in the training group compared with the control group. ^b^ *P* < 0.05 significant change from baseline to follow-up in the training group compared with the control group.

**Table 6:** Duration of each EMG gap (seconds) under 0.5% EMGmax for m. trapezius and m. splenius, median (interquartile range).

		Training (*N* = 15)	Control (*N* = 15)
Trapezius			
Before daily training session	Week 0	0.72 (0.6–1.74)	0.9 (0.54–1.68)
	Week 10	1.26 (0.6–1.98)^b^	0.6 (0.42–1.02)
After daily training session	Week 0	1.08 (0.66–1.8)	0.96 (0.6–1.38)
	Week 10	1.56 (0.72–2.76)	0.78 (0.36–1.26)
Splenius			
Before daily training session	Week 0	0.42 (0.36–0.48)	0.6 (0.48–0.96)
	Week 10	0.72 (0.54–0.78)^a^	0.36 (0.3–0.48)
After daily training session	Week 0	0.54 (0.42–0.6)	0.54 (0.42–0.72)
	Week 10	0.72 (0.54–1.02)	0.48 (0.42–0.54)

^a^ *P* < 0.01 significant change from baseline to follow-up in the training group compared with the control group. ^b^ *P* < 0.05 significant change from baseline to follow-up in the training group compared with the control group.
